# The EGCG and α-Mangosteen Stimulate SHED-IL10 and SHED-LL37 Metabolite Concentration

**DOI:** 10.1055/s-0043-1761460

**Published:** 2023-04-14

**Authors:** Yuliati Yuliati, Fatma Yasmin Mahdani, Sellyn Angelina Margaretha, Wahidah Tsamara Putri Yastuti, Meircurius Dwi Condro Surboyo, Mohammed Ahmed Aljunaid, Huda Rashad Qaid, Rini Devijanti Ridwan, Indeswati Diyatri

**Affiliations:** 1 Doctoral Program, Faculty of Dental Medicine, Universitas Airlangga, Surabaya, Indonesia; 2 Department of Oral Biology, Faculty of Dental Medicine, Universitas Airlangga, Surabaya, Indonesia; 3 Oral Medicine Study Program, Faculty of Dental Medicine, Universitas Airlangga, Surabaya, Indonesia; 4 Department of Oral Medicine, Faculty of Dental Medicine, Universitas Airlangga, Surabaya, Indonesia; 5 Bachelor of Dental Science Program. Faculty of Dental Medicine, Universitas Airlangga, Surabaya, Indonesia; 6 Department of Oral and Dental Medicine, Faculty of Medicine, Taiz University, Taiz, Yemen; 7 Faculty of Oral and Dental Medicine, Al-Saeed University, Taiz, Yemen

**Keywords:** SHED, metabolite, EGCG, α-mangosteen, medicine

## Abstract

**Objective** Stem cells of human exfoliated deciduous teeth (SHED) metabolites are secreted molecules from SHED, namely cytokines, chemokines, and growth factors. The metabolite can be used in various regenerative therapy based on cell-free immunomodulatory potential effects, like interleukin 10 (IL-10) and LL37. This molecule can stimulate with epigallocatechin gallate (EGCG) and α-mangosteen and has been proven to have anti-inflammatory and antibacterial effects. This study aimed to identify the effect of EGCG and α-mangosteen to SHED metabolite, called SHED-IL10 and SHED-LL37, from six passages to obtain the optimum stimulation and able to use as periodontitis regeneration treatment.

**Materials and Methods** The six different passages of SHED were prepared in Dulbecco's modified Eagle medium and added with EGCG 80% (10 μM), EGCG 95% (10 μM), or α-mangosteen (10 μM). After a 24 hours incubation, each passage was measured with the metabolite concentration, SHED-IL10 and SHED-LL37, with human IL-10 and LL37 using enzyme-linked immunosorbent assay. Each different concentration was then analyzed statistically.

**Results** The addition of EGCG 95% is able to stimulate the SHED-IL10 optimum concentration in passage 1 ( *p* < 0.01). But, in the different conditions, the addition of EGCG 80%, EGCG 95%, and α-mangosteen was able to stimulate the SHED-LL37 optimum concentration in passage 2 ( *p* < 0.001).

**Conclusion** The addition of EGCG and α-mangosteen can stimulate the SHED-IL10 and SHED-LL37 concentrations. These two metabolites are promising as regenerative therapy through anti-inflammatory and antibacterial properties.

## Introduction

Stem cells of human exfoliated deciduous teeth (SHED) are mesenchymal stem cells (MSC) with high differentiation potential, self-regeneration ability, and ease of obtaining. SHED is a source of stem cells and secreted various growth factors, cytokines and exosomes, known as metabolites and can be detected in stem cell culture media. 1 2 These secreted growth factors function as paracrine mediators for immunoregulation and tissue regeneration. Studies have revealed that the success of SHED-based therapy is likely to occur via a paracrine secretory mechanism. Important paracrine mediators include exosomes containing bioactive molecules, including proteins, lipids, signaling molecules, and mRNAs. Exosomes can act as nanocarriers to transfer bioactive molecules from stem cells to recipient cells and modulate recipient cell functions by secreting materials into target cells as communication signals via ligand or receptor molecules on the surface or by fusion of exosomes with cell membranes. 3 4

The epicatechin gallate (EGCG), the main component of polyphenols in green tea, plays an essential role as an antioxidant, antitumor, anti-inflammatory, and antimicrobial properties. Several studies of EGCG stimulate the differentiation of stem cells in bone mesenchymal tissue and increase the formation of the periodontal ligament. 5 However, EGCG possesses protection properties for bone health, reducing bone resorption through the antioxidant, anti-inflammatory, suppressing osteoclastogenesis, and osteoimmunological effects. The EGCG has anti-inflammatory properties through its ability to scavenge nitric oxide (NO), peroxynitrite, reactive oxygen species, reactive nitrogen species, cyclooxygenase, interleukins (ILs), and tumor necrosis factor (TNF-α) in activated macrophages. 6 7 8 Not only the EGCC, but some of the plants also contain an active substance that possesses antioxidant, antiallergic, anti-inflammatory, antibacterial, antifungal, antitumor, and antiviral properties called α-mangosteen. By these properties, the α-mangosteen can inhibit prostaglandin E2 synthesis, IL synthesis, and TNF-α, like an EGCG. 9

The previous study of the EGCG and α-mangosteen in periodontitis has developed. The finding showed that EGCG inhibits the process of alveolar bone damage through RANKL, 10 TNF-α, 11 and increases osteoprotegerin (OPG), 10 RANK, 10 11 and IL-10. 11 12 The effect is similar to α-mangosteen, which is able to stimulate some growth factors like tumor growth factor-β, 13 and TRAP5b 14 and also inhibit the growth of etiological bacteria 15 16 and RUNX2. 14

Periodontitis is a disease of the periodontal tissues that is characterized by damage to the ligaments and surrounding alveolar bone. This disease can be caused by several bacteria, including *Porphyromonas gingivalis* , and in severe periodontitis, it can cause tooth loss. 17 Periodontitis therapy is focused on inhibiting bone resorption by stimulating the anti-inflammatory mediator, such as IL-10, that inhibits bone resorption. 18 The other antimicrobial protein that is needed is LL37, which has antimicrobial properties through autophagy of *Porphyromonas gingivalis* as the etiology of periodontitis 19 and can suppress the production of proinflammatory cytokines. 20 Due to the various properties of EGCG and α-mangosteen, especially the anti-inflammatory properties, this study aimed to identify the effect of EGCG and α-mangosteen to SHED metabolite, called SHED-IL10 and SHED-LL37, from six passages to obtain the optimum stimulation and able to use as periodontitis regeneration treatment.

## Materials and Methods

### Study Design

The study design is a valid analytical, experimental laboratory; the protocol was approved by the Ethical Health Committee of the Faculty of Dental Medicine, Universitas Airlangga, Surabaya, Indonesia, with number 834/HRECC.FODM/XI/2022.

### SHED Metabolites

SHED metabolites are purified from the SHED provided by the Research Centre Faculty of Dental Medicine Universitas Airlangga. The SHED was cultured from passages 1 to 6 in Dulbecco's modified Eagle medium. SHED culture medium was purified using the dialysis method to remove waste products of metabolism that were not useful, resulting in beneficial results of metabolites that contained several cytokines, growth factors, and exosomes.

### EGCG and α-Mangosteen

The EGCG used in this experiment was two different types: EGCG 98% (epigallocatechin-gallate, Chamfaces, Wuhan, China) and EGCG 80% (Medi tea, Dharma Putra Airlangga, Surabaya, Indonesia). The α-mangosteen concentration 98% (M3824, Sigma Aldrich, Merck, Germany).

### SHED-IL-6 and LL37 Concentration

The SHED-IL10 and LL37 concentrations were measured using enzyme-linked immunosorbent assay during passages one and six. Before the measurement, each well-contained SHED metabolite was added by 10 μM EGCG 98%, 10 μM EGCG 80%, or 10 μM α-mangosteen and incubated for 24 hours. The antibody used was human IL-10 (human IL-10, BT Laboratory, Shanghai, China) and human LL37 (human LL317, IL-10, BT Laboratory, Shanghai, China). The SHED-IL10 and LL37 were immediately measured (three replication) by the optical density value of each well using a microplate reader set to 450 nm.

### Statistical Analysis

The differences in SHED-IL10 and LL37 concentration in each passage were analyzed using one-way analysis of variance and posthoc test Tukey HSD. The Statistical Package for Social Science (SPSS) version 29.0 for Mac (IBM Corporation, Chicago, Illinois, United States) was used to analyze the data.

## Results

### SHED-IL10 and SHED-LL37 Concentration

**Fig. 1:**
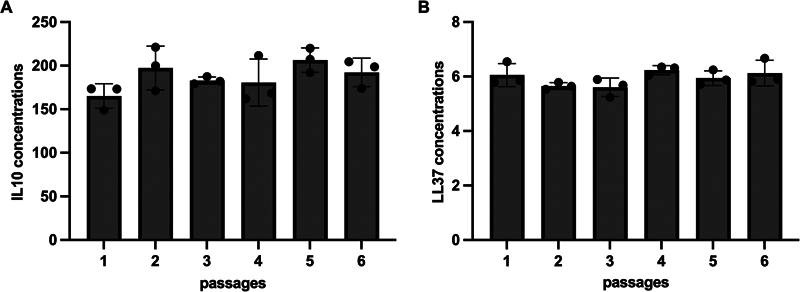
The SHED-IL10 ( **A** ) and SHED-LL37 ( **B** ) concentration on the basic condition.

The SHED-IL10 and SHED-LL37 concentrations are presented in Fig. 1 . The highest SHED-IL10 concentration on the basic condition was observed in passage 5, while the SHED-LL37 concentration was observed in passage 4.

#### The Addition of EGCG or α-Mangosteen on SHED-IL10 Concentration

**Fig. 2:**
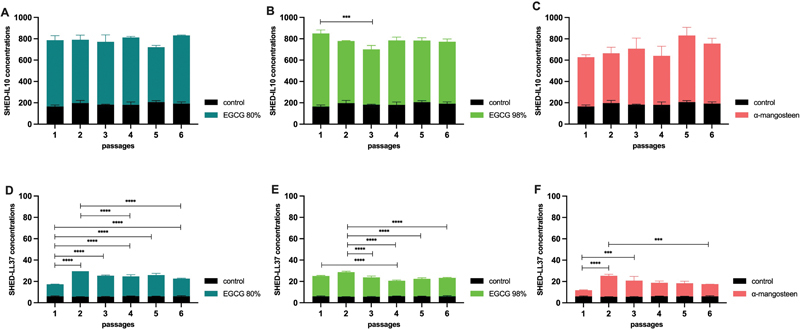
The SHED-IL10 ( **A** – **C** ) and SHED-LL37 ( **E** – **F** ) concentrations by two different epigallocatechin gallate (EGCG) concentrations and α-mangosteen. *** *p* < 0.001; **** *p* < 0.0001.

The addition of EGCG 80% and α-mangosteen did not show a SHED-IL10 concentration in the different passages ( Fig. 2A–C ). On the other hand, the addition of EGCG 95% showed a higher SHED-IL10 concentration in passage 1 than in passage 3 ( *p* < 0.001; Fig. 2B ).

#### The Addition of EGCG or α-Mangosteen on SHED-LL37 Concentration

The addition of EGCG 80%, EGCG 95%, and α-mangosteen showed a difference in SHED-LL37 concentration in every passage. The addition of EGCG 80% showed a higher SHED-LL37 in passage 2 than in passage 1, passage 4 and passage 6 ( *p* < 0.0001; Fig. 2D ). A similar condition is also observed in the addition of EGCG 95%. The higher SHED-LL37 was observed in passage 2 than in passage 3 until 6 ( *p* < 0.0001; Fig. 2E ). The addition of α-mangosteen showed a higher SHED-LL37 concentration in passage two than in passage 1 ( *p* < 0.001) and passage 6 ( *p* < 0.0001; Fig. 2F ).

## Discussion

Stem cell metabolites must be validated before use. The validation process includes an assessment of potential, which shows the ability of stem cells to differentiate, and purity to prove that these cells are actual stem cells. Stem cell metabolites are also characterized *in vitro* and *in vivo* before finally being applied to humans, not the exception of SHED. 21 22 23 24

In this study, the addition of EGCG 80%, EGCG 95%, and α-mangosteen was able to stimulate the SHED metabolite called SHED-IL10. The stimulation is probably due to the decreased expression of inflammatory genes. Another study showed that the addition of α-mangosteen increased the number of IL-10-producing T cells and IL-10 gene expression in 7F2 osteoblast cell culture with osteogenic media. It is suspected that α-mangosteen is an immunomodulator by stabilizing or directly exerting anti- or proinflammatory activity. IL-10, a cytokine with pleiotropic immunosuppressive function, is also a founding member of the IL-10 cytokine family. IL-10 is an early feature of the picture as an inhibitory factor for the synthesis of secreted cytokines produced by T helper (Th) 2 cell clones with the ability to inhibit Th1 cytokine production. Subsequently, IL-10 was reported to be expressed by various cell types in the immune system's innate and adaptive arms. 25 26 IL-10 mainly targets antigen-presenting cells, such as monocytes and macrophages, and inhibits the release of proinflammatory cytokines, such as TNF-α, IL-1β, IL-6, 27 28 granulocyte colony-stimulating factor, and granulocyte-macrophage colony-stimulating factor. 12 26 29 The IL-10 has an important role in periodontitis, 30 which is able to control the number of bacteria, 31 regulation of pro-inflammatory cytokine, and increase OPG and osteoclastogenesis. 32 The previous study by Aljunaid et al and Lashari et al also showed that the EGCG is able to stimulate the IL-10 expression 11 12 and exhibit the RANKL as an osteoclast marker. 10 As previously mentioned, IL-10 suppresses several key proinflammatory cytokines that are clinically validated to participate in the pathogenesis of any disease of the oral mucosa, especially periodontitis. 12

Not only stimulated the SHED-IL10, but the addition of EGCD and α-mangosteen also stimulated the SHED-LL37 concentration. LL37, a class of antimicrobial peptides, is the sole member of the human cathelicidin family, produced by many cell types, including macrophages, natural killer cells, skin epithelial cells, airways, mucosal, ocular surface, and intestine. 33 This peptide piqued the research community's interest because it carries numerous immune systems modulating and antimicrobial properties. 34 The antimicrobial properties through pore formation in gram-positive and gram-negative bacteria and neutralize LPS. 35 36 And by that, the LL37 is able to express receptors that to pathogen-associated molecular patterns, cooperate with human-β-defensin-2, 33 34 and actively promote leukocyte recruitment to the area of infection. 37 38 In monocytes, LL37 significantly inhibited the expression of NF-kB, TNF-α, and NO produced by LPS and macrophages. 28 37 38 The role of LL37 in periodontitis is related to its antimicrobial properties, 39 inhibits osteoblast apoptosis, 40 and modulates gingival fibroblast proliferation. 41 The same result was also found in Li et al study regarding human adipose-derived mesenchyme stem cells (hADSCs) which showed that LL37 could increase osteogenic differentiation *in vitro* as well as antibacterial properties which play a role in the process of tissue regeneration in periodontal. 42

This study attempted to analyze the expression of IL-10 and LL37 on SHED. There have been no previous studies that have analyzed both markers. However, other studies have shown that stem cells are a source for producing or expressing IL-10, such as in MSCs. 43 44 With the important role of IL-10 and LL37 in periodontitis treatment or periodontal regeneration, it is essential to develop and increase the production through the tissue engineering process by SHED. This study showed that the addition of EGCG and α-mangosteen stimulated the optimum SHED-LL37 concentration in passage 2. But, the SHED-IL10 was able to obtain a maximum concentration in any passage. Future research needs to perform to calibrate this finding into tissue engineering models in animal and human studies.

## Conclusion

The result of this study provides evidence that the addition of EGCG and α-mangosteen can stimulate the SHED-IL10 and SHED-LL37 concentrations. These two metabolites are promising as regenerative therapy through anti-inflammatory and antibacterial properties. A future study needs to be performed to analyze this SHED metabolite's most significant anti-inflammatory and antibacterial properties in a different passage.

## References

[1] Wu J Chen L Wang R Exosomes secreted by stem cells from human exfoliated deciduous teeth promote alveolar bone defect repair through the regulation of angiogenesis and osteogenesis ACS Biomater Sci Eng 2019 5 07 3561 3571 33405738 10.1021/acsbiomaterials.9b00607

[2] Xie F He J Chen Y Hu Z Qin M Hui T Multi-lineage differentiation and clinical application of stem cells from exfoliated deciduous teeth Hum Cell 2020 33 02 295 302 32006349 10.1007/s13577-020-00323-z

[3] Nakao Y Fukuda T Zhang Q Exosomes from TNF-α-treated human gingiva-derived MSCs enhance M2 macrophage polarization and inhibit periodontal bone loss Acta Biomater 2021 122 306 324 33359765 10.1016/j.actbio.2020.12.046 PMC7897289

[4] Bar J K Lis-Nawara A Grelewski P G Dental Pulp Stem Cell-Derived Secretome and Its Regenerative Potential Int J Mol Sci 2021 22 21 12018 34769446 10.3390/ijms222112018 PMC8584775

[5] Liu Z Lin Y Fang X Yang J Chen Z Epigallocatechin-3-gallate promotes osteo-/odontogenic differentiation of stem cells from the apical papilla through activating the BMP–SMAD signaling pathway Molecules 2021 26 06 1580 33809391 10.3390/molecules26061580 PMC8001198

[6] Novilla A Djamhuri D S Nurhayati B Rihibiha D D Afifah E Widowati W Anti-inflammatory properties of oolong tea (Camellia sinensis) ethanol extract and epigallocatechin gallate in LPS-induced RAW 264.7 cells Asian Pac J Trop Biomed 2017 7 11 1005 1009

[7] Mah Y J Song J S Kim S O The effect of epigallocatechin-3-gallate (EGCG) on human alveolar bone cells both in vitro and in vivo Arch Oral Biol 2014 59 05 539 549 24637009 10.1016/j.archoralbio.2014.02.011

[8] Rahmanisa S Oktaria R Pengaruh epigallocatechin-3-gallate (EGCG) pada the hijau terhadap Acne vulgaris Jurnal Majority 2016 5 02 101 105

[9] Jaisupa N Moongkarndi P Lomarat P Mangosteen peel extract exhibits cellular antioxidant activity by induction of catalase and heme oxygenase-1 mRNA expression J Food Biochem 2018 42 e12511

[10] Lashari D M Aljunaid M Lashari Y The use of mucoadhesive oral patches containing epigallocatechin-3-gallate to treat periodontitis: an in vivo study J Taibah Univ Med Sci 2022 17 06 1014 1020 36212598 10.1016/j.jtumed.2022.06.006 PMC9519795

[11] Aljuanid M A Qaid H R Lashari D M Nano-emulsion of mangosteen rind extract in a mucoadhesive patch for periodontitis regenerative treatment: an in vivo study J Taibah Univ Med Sci 2022 17 05 910 920 36050950 10.1016/j.jtumed.2022.03.003 PMC9396070

[12] Lashari D M Aljunaid M A Ridwan R D The ability of mucoadhesive gingival patch loaded with EGCG on IL-6 and IL-10 expression in periodontitis J Oral Biol Craniofac Res 2022 12 05 679 682 36062255 10.1016/j.jobcr.2022.08.007 PMC9434042

[13] Lestari C Darwin E Putra D P Suharti N The α-mangostin effect on the quantity of TGF-β1 titer relate to the mandibular bone volume of Rattus novergicus in the periodontitis model J Pharm Pharmacogn Res 2021 9 05 609 618

[14] Lestari C Darwin E Putra D P Suharti N The Effect of α- *Mangosteen* on Runt-Related Transcription Factor 2 and Tartrate-Resistant Acid Phosphatase 5b Expressions on Bone Remodeling in Periodontitis (An Experimental Research on Wistar Rats) In: Proceedings o ^f^ 1st International Conference on Health Sciences and Biotechnology (ICHB 2021) [Internet]. 2022. Accessed January 14, 2023 at: https://www.atlantis-press.com/article/125971184

[15] Torrungruang K Vichienroj P Chutimaworapan S Antibacterial activity of mangosteen pericarp extract against periodontal pathogens J Dent Assoc Thai 2007 57 05 240 246

[16] Tangsuksan P Srichana T Kettratad M Nittayananta W Antimicrobial and anti-inflammatory effects of α-mangosteen soluble film J Int Soc Prev Community Dent 2022 12 02 189 198 35462748 10.4103/jispcd.JISPCD_222_21 PMC9022392

[17] Zhang Z Liu D Liu S Zhang S Pan Y The role of Porphyromonas gingivalis outer membrane vesicles in periodontal disease and related systemic diseases Front Cell Infect Microbiol 2021 10 585917 33585266 10.3389/fcimb.2020.585917 PMC7877337

[18] Zhang Q Chen B Yan F Interleukin-10 inhibits bone resorption: a potential therapeutic strategy in periodontitis and other bone loss diseases BioMed Res Int 2014 2014 284836 24696846 10.1155/2014/284836 PMC3947664

[19] Yang X Niu L Pan Y LL-37-induced autophagy contributed to the elimination of live *Porphyromonas gingivalis* internalized in keratinocytes Front Cell Infect Microbiol 2020 10 561761 33178622 10.3389/fcimb.2020.561761 PMC7593823

[20] Ribeiro A ERA Lourenço A G Motta A CF Komesu M C Salivary Expression of antimicrobial peptide LL37 and its correlation with pro-inflammatory cytokines in patients with different periodontal treatment needs Int J Pept Res Ther 2020 26 04 2547 2553

[21] Sumorejo P Listiawan M Y Putri A I Rantam F A Susilowati H Hendrianto E The role of stem cell metabolites derived from placenta for skin regeneration: an in vitro study Bali Med J 2019 8 01 354 359

[22] Citterio F Gualini G Fierravanti L Aimetti M Stem cells and periodontal regeneration: present and future Plast Aesthet Res 2020 7 41

[23] Kim S H Seo B M Choung P H Lee Y M Adult stem cell therapy for periodontal disease Int J Stem Cells 2010 3 01 16 21 24855536 10.15283/ijsc.2010.3.1.16 PMC4022685

[24] Xia J Minamino S Kuwabara K Arai S Stem cell secretome as a new booster for regenerative medicine Biosci Trends 2019 13 04 299 307 31527327 10.5582/bst.2019.01226

[25] Zhang Q Chen B Yan F Interleukin-10 inhibits bone resorption: A potential therapeutic strategy in periodontitis and other bone loss diseases Biomed Res Int 2014 2014 284836 24696846 10.1155/2014/284836 PMC3947664

[26] Shi T Jin Y Miao Y Wang Y Zhou Y Lin X IL-10 secreting B cells regulate periodontal immune response during periodontitis Odontology 2020 108 03 350 357 31701299 10.1007/s10266-019-00470-2

[27] Gutierrez-Orozco F Chitchumroonchokchai C Lesinski G B Suksamrarn S Failla M L α-Mangosteen: anti-inflammatory activity and metabolism by human cells J Agric Food Chem 2013 61 16 3891 3900 23578285 10.1021/jf4004434 PMC3793015

[28] Sayekti M Rizqiawan A Soesilawati P Mira N P Kamadjaja D B Rahman M Z The effect of alpha-mangosteen on interleukin-10 and collagen 1A1 gene in the inflammation process: an experimental in-vitro study J Int Oral Health 2022 14 04 403 408

[29] Wang X Wong K Ouyang W Rutz S Targeting IL-10 family cytokines for the treatment of human diseases Cold Spring Harb Perspect Biol 2019 11 02 a028548 29038121 10.1101/cshperspect.a028548 PMC6360861

[30] Toker H Gorgun E P Korkmaz E M Yüce H B Poyraz O The effects of IL-10 gene polymorphism on serum, and gingival crevicular fluid levels of IL-6 and IL-10 in chronic periodontitis J Appl Oral Sci 2018 26 00 e20170232 29489938 10.1590/1678-7757-2017-0232 PMC5829549

[31] Geng Y Li L Wang X Interleukin-10 polymorphisms affect the key periodontal pathogens in Chinese periodontitis patients Sci Rep 2018 8 01 9068 29899423 10.1038/s41598-018-26236-4 PMC5997982

[32] Chen X Wan Z Yang L Exosomes derived from reparative M2-like macrophages prevent bone loss in murine periodontitis models via IL-10 mRNA J Nanobiotechnology 2022 20 01 110 35248085 10.1186/s12951-022-01314-y PMC8898524

[33] Kahlenberg J M Kaplan M J Little peptide, big effects: the role of LL-37 in inflammation and autoimmune disease J Immunol 2013 191 10 4895 4901 24185823 10.4049/jimmunol.1302005 PMC3836506

[34] Pahar B Madonna S Das A Albanesi C Girolomoni G Immunomodulatory Role of the Antimicrobial LL-37 Peptide in Autoimmune Diseases and Viral Infections Vaccines 2020 8 03 517 32927756 10.3390/vaccines8030517 PMC7565865

[35] Rosenfeld Y Papo N Shai Y Endotoxin (lipopolysaccharide) neutralization by innate immunity host-defense peptides. Peptide properties and plausible modes of action J Biol Chem 2006 281 03 1636 1643 16293630 10.1074/jbc.M504327200

[36] Kim H J Cho D H Lee K J LL-37 suppresses sodium nitroprusside-induced apoptosis of systemic sclerosis dermal fibroblasts Exp Dermatol 2011 20 10 843 845 21732986 10.1111/j.1600-0625.2011.01327.x

[37] Alcayaga-Miranda F Cuenca J Khoury M Antimicrobial activity of mesenchymal stem cells: current status and new perspectives of antimicrobial peptide-based therapies Front Immunol 2017 8 339 28424688 10.3389/fimmu.2017.00339 PMC5371613

[38] Chessa C Bodet C Jousselin C Wehbe M Lévêque N Garcia M Antiviral and immunomodulatory properties of antimicrobial peptides poduced by human keratinocytes Front Microbiol 2020 11 1155 32582097 10.3389/fmicb.2020.01155 PMC7283518

[39] Chinipardaz Z Zhong J M Yang S Regulation of LL-37 in bone and periodontium regeneration Life (Basel) 2022 12 10 1533 36294968 10.3390/life12101533 PMC9604716

[40] Chen K Gong W Huang J Yoshimura T Wang J M The potentials of short fragments of human anti-microbial peptide LL-37 as a novel therapeutic modality for diseases Front Biosci 2021 26 11 1362 1372 10.52586/5029 34856773

[41] McCrudden M TC O'Donnell K Irwin C R Lundy F T Effects of LL-37 on gingival fibroblasts: a role in periodontal tissue remodeling? Vaccines (Basel) 2018 6 03 44 30041453 10.3390/vaccines6030044 PMC6161023

[42] Li L Peng Y Yuan Q Sun J Zhuang A Bi X Cathelicidin LL37 promotes osteogenic differentiation *in vitro* and bone regeneration *in vivo* Front Bioeng Biotechnol 2021 9 638494 34012955 10.3389/fbioe.2021.638494 PMC8126666

[43] Zhang C Delawary M Huang P Korchak J A Suda K Zubair A C IL-10 mRNA engineered MSCs demonstrate enhanced anti-inflammation in an acute GvHD model Cells 2021 10 11 3101 34831324 10.3390/cells10113101 PMC8621791

[44] Wang J Ren H Yuan X Ma H Shi X Ding Y Interleukin-10 secreted by mesenchymal stem cells attenuates acute liver failure through inhibiting pyroptosis Hepatol Res 2018 48 03 E194 E202 28833919 10.1111/hepr.12969

